# Case Report: Phototherapeutic Keratectomy for Band Keratopathy Secondary to Chemo-Laser-Cryotherapy for Retinoblastoma

**DOI:** 10.3389/fmed.2021.668762

**Published:** 2021-08-25

**Authors:** Ruoyan Wei, Meiyan Li, Weiming Yang, Haipeng Xu, Joanne Choi, Xingtao Zhou

**Affiliations:** ^1^Department of Ophthalmology and Optometry, Eye and ENT Hospital, Fudan University, Shanghai, China; ^2^NHC Key Laboratory of Myopia (Fudan University), Shanghai, China; ^3^Shanghai Research Center of Ophthalmology and Optometry, Shanghai, China; ^4^Department of Ophthalmology, Children's Hospital of Fudan University, Shanghai, China; ^5^Kresge Eye Institute/Department of Ophthalmology, Wayne State University, Detroit, MI, United States

**Keywords:** phototherapeutic keratectomy (PTK), band keratopathy, retinoblastoma, treatment, visual outcome

## Abstract

**Purpose:** To report the clinical outcomes of phototherapeutic keratectomy (PTK) for pediatric band keratopathy after treatment for retinoblastoma.

**Methods:** A 5-year-old boy presented with a 2-year history of poor visual acuity and a horizontal gray-white band across the central cornea in the right eye. He was diagnosed with band keratopathy after chemo-laser-cryotherapy for retinoblastoma. The band keratopathy was treated via PTK using the Mel-90 excimer laser with an optical treatment zone of 7.0 mm and ablation depth of 120 μm. The patient was followed at 1 week and 3 months postoperatively.

**Results:** Surgery and postoperative follow-up were uneventful. At the 3-month follow-up, the uncorrected distant visual acuity of the right eye improved to 20/125, and the corrected distance visual acuity improved to 20/70 with a refraction of +10.00 D/−2.50 DC × 15. The clarity of the ablated area was evidently improved. The central corneal thickness decreases from 612 to 584 μm. The optical coherence tomography showed the thin band of hyperreflectivity in the ablated area disappeared, corneal transparency improved and the corneal surface smoothened.

**Conclusions:** PTK is a safe and effective procedure to treat band keratopathy following treatment of retinoblastoma in children. Early intervention can reduce the risk of developing deprivation amblyopia.

## Introduction

Retinoblastoma is a severe pediatric intraocular malignancy. Though the survival rates have increased dramatically, vision may be negatively affected by the potential complications of focal therapy and radiotherapy, such as non-axial cataract and keratopathy (1–4).

Band keratopathy is a chronic degenerative disease characterized by the deposition of gray-white opacities in the sub-epithelial cornea. It is associated with systemic hypercalcemia and ocular inflammatory disease (5) such as uveitis, and is also a common complication of local chemotherapy and radiation (6). This case documents the management of band keratopathy secondary to retinoblastoma treatment using phototherapeutic keratectomy (PTK) and reports on its safety and efficacy.

## Case Report

A 5-year-old boy presented on November 6th, 2019 with a 2-year history of gradually worsening vision and a growing horizontal gray-white opacity across the central cornea in his right eye.

His ocular history is significant for bilateral retinoblastoma first diagnosed at 10 months of age. At 9 months of age, leukocoria was first observed in his left eye, and he was diagnosed with bilateral retinoblastoma 1 month later. He was treated systemically with 8 cycles of vincristine, etoposide, and carboplatin (VEC) systemic chemotherapy and also received intravitreal melphalan chemotherapy, laser photocoagulation, and cryotherapy in both eyes as well as transpupillary thermotherapy in the left eye. After 8 months of treatment, tumor reduction was achieved and there were no signs of recurrence.

**Figure 1:**
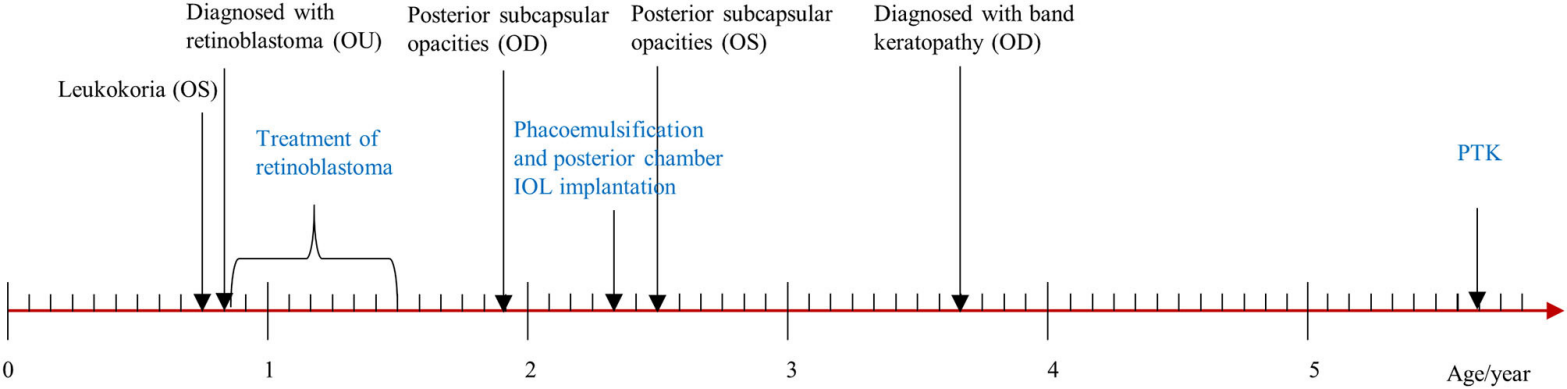
Timeline of disease and treatment periods. OU, both eyes; OD, right eye; OS, left eye; IOL, intraocular lens; PTK, phototherapeutic keratectomy.

Five months after the conclusion of treatment for retinoblastoma, the patient developed posterior sub-capsular opacities in the right eye and underwent phacoemulsification cataract extraction and implantation of a +20.0 D intraocular lens. Twelve months after retinoblastoma treatment, posterior sub-capsular opacities were also detected in the left eye. Besides, twenty-six months' post-treatment, during a routine follow-up visit for retinoblastoma, a horizontal gray-white opacity on the surface of his right eye was noted. Based on slit lamp exam findings, it was diagnosed as band keratopathy, however, no intervention was attempted at this time (Figure 1).

Examination revealed an uncorrected distance visual acuity (UDVA) of hand movement OD (right eye) and count fingers at 40 cm OS (left eye). The intraocular pressure (IOP) as measured by non-contact tonometer was 6.5 mmHg OD and 7.5 mmHg OS. Slit-lamp examination showed a 3 mm × 8 mm band-shaped, gray-white sub-epithelial corneal opacity across the horizontal axis in the right eye. The cornea of the left eye was clear.

**Figure 2:**
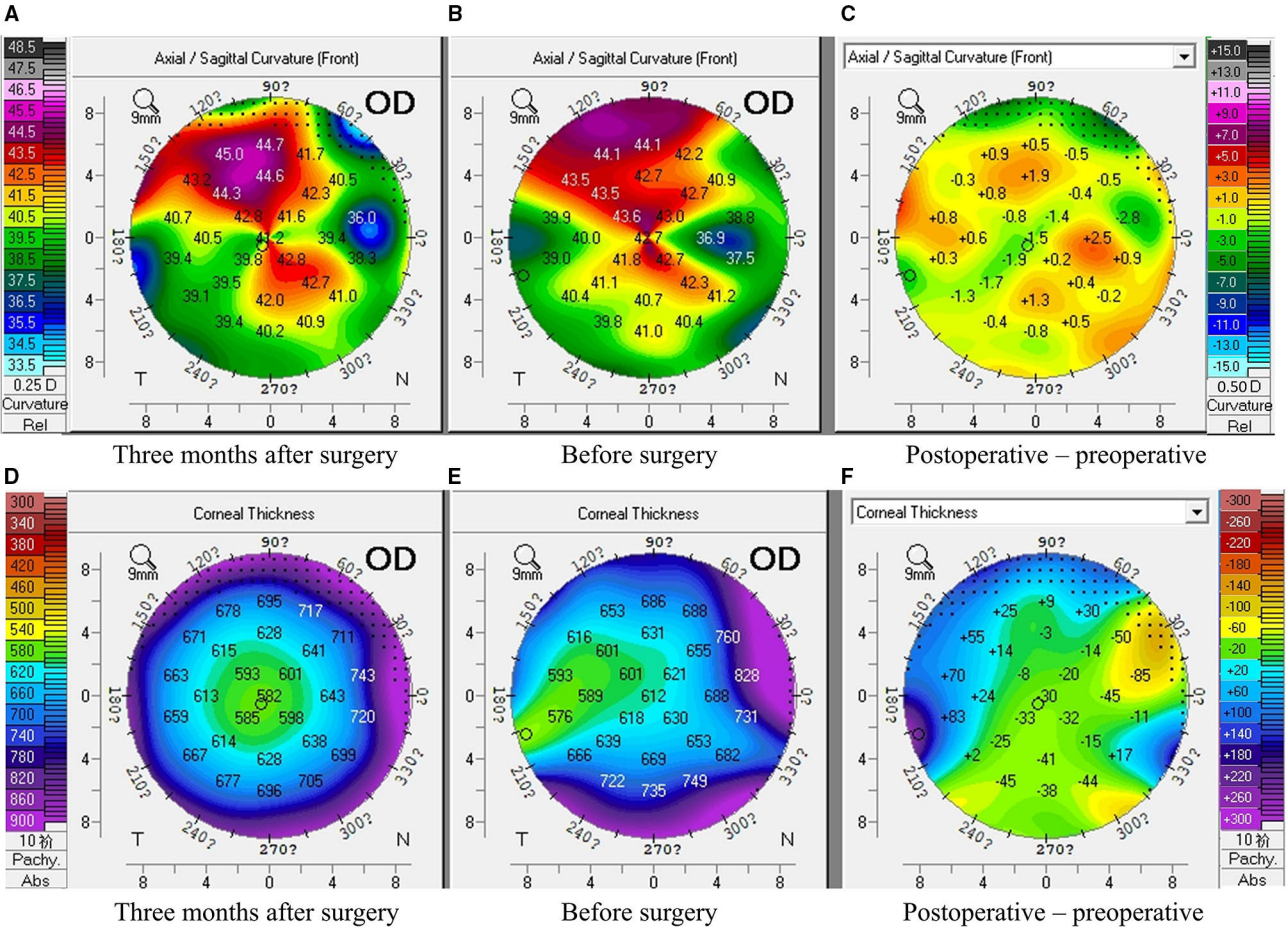
Corneal topography of band keratopathy following treatment of retinoblastoma before and three months after surgery. **(A–C)** The corneal curvature remained stable pre and post-operation (**A**: three months after surgery, **B**: before surgery, **C**: the difference between a and **B**); **(D,E)** The corneal thickness decreased after the surgery (**D**: three months after surgery, **E**: before surgery, **F**: the difference between **D** and **E**).

**Figure 3:**
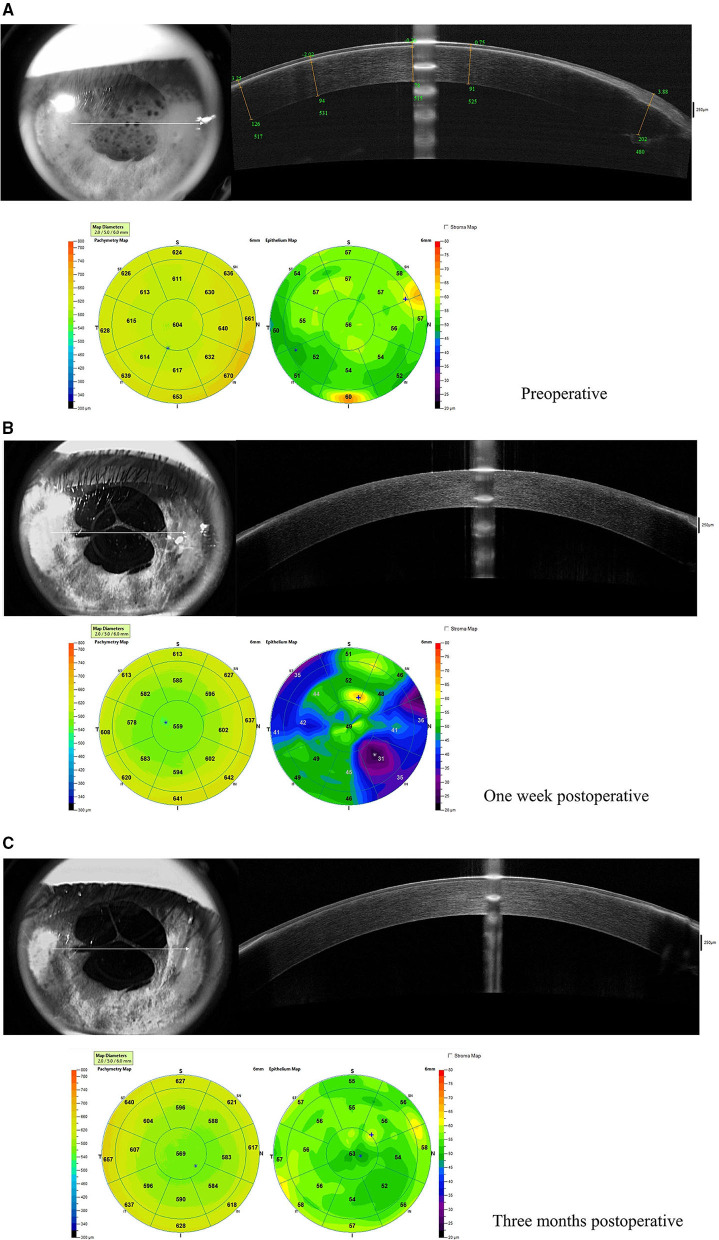
Anterior segment optical coherence tomography (AS-OCT) before and after surgery. **(A)** Before surgery, a thin band of hyperreflectivity was along with Bowman's layer. The upper right panels show the five segments (yellow lines) on the horizontal meridian. The green numbers represent the distance from the corneal vertex, the depth of the band, and the corneal thickness at the specific site, respectively; **(B)** one week and **(C)** three months after surgery, the band of hyperreflectivity disappeared.

The corneal topography of the right eye was evaluated with Pentacam (Oculus Optikgeräte GmbH, Wetzlar, Germany) (Figure 2) and revealed a central corneal thickness of 612 μm and mean anterior keratometry of 41.1 D. Anterior segment optical coherence tomography (AS-OCT) showed a thin band of hyperreflectivity involving Bowman's layer with underlying shadowing (Figure 3A).

Twenty-four months after diagnosis, the patient's family elected to treat the band keratopathy and PTK was performed under topical anesthesia using the Mel-90 excimer laser with a 250 Hz pulse rate and 1 mJ pulse energy (Carl Zeiss Meditec, Jena, Germany). The optical treatment zone was set to 7.0 mm, and the ablation depth was set to 120 μm according to the measurement of AS-OCT. No topography-guided ablation nor masking agent was applied. Postoperative care consisted of a bandage silicone hydrogel soft contact lens (ACUVUE OASYS, Inc., FL, USA) and topical levofloxacin 4 times per day for 1 week, topical 0.1% fluorometholone solution tapered from 8 times daily to 1 time daily over 2 months, and preservative-free artificial tears 4 times daily for 1 month. This study adhered to the Declaration of Helsinki and was approved by the Ethics Committee of the Fudan University EENT Hospital Review Board (No. 2017060). The surgery was uneventful, and no intraoperative or postoperative complications (such as infection, sub-epithelial fibrosis, or haze) were noted. The surgery was well-tolerated by the patient and he reported minimal pain and discomfort both during and after the surgery.

**Figure 4:**
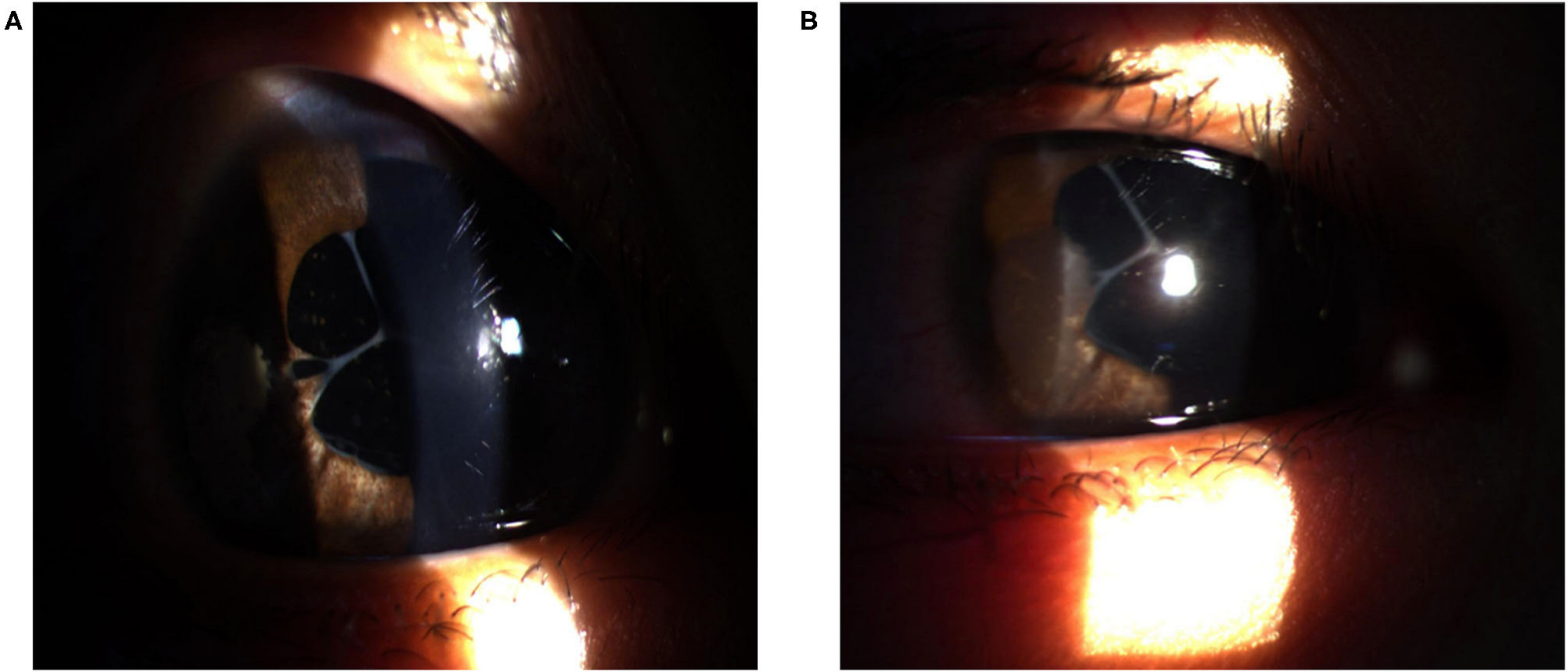
Slit-lamp image of the cornea after surgery. The optical zone was clear and transparent after 1 week **(A)** and 3 months **(B)**.

Postoperatively at 1-week, UDVA in the right eye was improved to 0.90 LogMAR (20/160). Manifest refraction was +11.00 DS/−1.00DC × 165 and corrected distance visual acuity (CDVA) was 0.80 LogMAR (20/125). Slit-lamp examination showed improved clarity of the ablated area (Figure 4A). AS-OCT showed the thin band of hyperreflectivity in the ablated area had disappeared, corneal transparency had improved and the corneal surface had smoothened (Figure 3B).

At a 3-month postoperative PTK follow-up, the UDVA in the right eye was further improved to 0.80 LogMAR (20/125) and manifest refraction was +10.00 DS/−2.50DC × 15 and CDVA was 0.54 LogMAR (20/70). The axial length of the right eye was 20.41 mm (IOL Master; Carl Zeiss Meditec). Slit-lamp examination showed that the clarity of the ablated area was clear (Figure 4B). The corneal curvature remained stable while the corneal thickness decreased after the surgery (Figure 2). In AS-OCT, no band of hyperreflectivity was observed in the ablated area, and the cornea remained transparent and smooth (Figure 3C). The consent for publication in print has been obtained from the patient.

## Discussion

Excimer laser PTK is a surgery used extensively to remove superficial corneal opacities and attain smooth and regular corneal surfaces, thereby improving visual acuity. A common indication for PTK is recurrent corneal erosion syndrome and anterior stromal and superficial scarring (7). This procedure has been proven to be safe and effective in both adults (8, 9) and children ranging from 8 to 18 years (10). Kollias et al. reported on the efficacy of PTK in five children with dense superficial corneal opacity (11). The efficacy and safety of PTK in treatment of band keratopathy was also investigated (12–15). O'Brart et al. reported that after PTK, ocular discomfort was improved in 95% of patients with band keratopathy and visual acuity was increased in 88% of patients (12). Nascimento et al. assessed outcomes after PTK in 7 children and 5 adults with band keratopathy secondary to chronic uveitis and indicated that all eyes of children had visual improvement and the improvement was more significant than that in adults (14). However, there have been no reports on PTK for the treatment of band keratopathy secondary to retinoblastoma treatment. In such complicated cases, the safety and efficacy of PTK is worth addressing and this case report aims to do so.

In this case report, the patient's visual acuity improved after PTK. This improvement was corroborated by AS-OCT which showed increased corneal transparency and a smoother corneal surface. Additionally, there were no postoperative complications such as infection, sub-epithelial fibrosis, or haze. Traditionally, manual superficial keratectomy and ethylenediaminetetraacetic acid (EDTA) chelation is used for removal of the calcific deposits with good efficacy (16); however, complete removal of deeply impacted or EDTA-resistant deposits was difficult. Manual error can also be hardly avoided and relatively imprecise tissue removal might lead to trauma to adjacent tissue. Further, after manual superficial keratectomy, 18% of eyes have been reported to have sub-epithelial haze (17). In comparison, PTK could precisely remove corneal deposits without manual error. Admittedly, the excimer laser ablation does not discriminate between calcium and normal tissue; therefore, when the calcific deposits are not uniform, it may leave an irregular surface (16). Application of masking agent, such as balanced salt solution, could avoid irregular ablation. To be clear, the corneal curvature remained stable pre and post operation and the postoperative spherical error of +10.00 D was more likely to be caused by the undercorrection of IOL power (axial length: 20.41 mm, IOL power: +20.0 D) rather than the PTK procedure. The spectacles or contact lenses was required to correct his refractive error. Therefore, PTK, a procedure that removes diseased tissue with extreme precision and minimal collateral damage, could be a good treatment option.

Band keratopathy after the treatment of retinoblastoma as described in this case is not rare in clinical practice though it is little reported. Retinoblastoma remains the most common primary intraocular malignancy of childhood and its incidence worldwide is around 1:15,000–1:20,000 live births with 11 new cases per million in children under 5 years old (18). A variety of treatment options are available, including local and systemic chemotherapy, cryotherapy, laser photoablation, radioactive plaques, external-beam radiation therapy, and enucleation (19). However, the side effects of such therapies, i.e., cataracts and keratopathies (1–4), can leave eyes with poor visual function if left untreated. The visual pathways develop from birth to approximately 6–8 years of age. Therefore, band keratopathy which occurs in early childhood may disrupt visual cortex development, leading to deprivation amblyopia and causing a progressive reduction of visual acuity. Early intervention with PTK could improve visual rehabilitation and reduce the risk of developing deprivation amblyopia.

To the best of our knowledge, this is the youngest reported patient (age 5 years old) to have undergone PTK with only surface anesthesia and no sedation. To overcome potential issues such as uncooperative eye movement during the procedure, we used educational training preoperatively to increase his familiarity with staff and equipment as well as to alleviate his stress and anxiety. During the procedure, two surgical forceps were used to help to fix the eye. Additionally, the cooperation between members of the surgical team was essential.

The findings of this case indicated that PTK could be an effective treatment for band keratopathy after retinoblastoma chemo-laser-cryotherapy. A longer follow-up would be helpful to evaluate its safety and impact on sustained vision recovery in young children.

## Value Statement

### What Was Known

1. Phototherapeutic keratectomy (PTK) is safe and effective to remove superficial corneal opacities and attain smooth and regular corneal surfaces, thereby improving visual acuity.

### What This Paper Adds

1. This is the youngest reported patient (age 5 years old) to have undergone PTK with only surface anesthesia and no sedation. The surgery was uneventful and was shown safe and effective during the follow-up.
2. The band keratopathy in this complicated case was secondary to retinoblastoma treatment. The early intervention with PTK could improve the prognosis for vision recovery and reduce the risk of amblyopia.

## Data Availability Statement

The original contributions presented in the study are included in the article/supplementary material, further inquiries can be directed to the corresponding author/s.

## Ethics Statement

The studies involving human participants were reviewed and approved by Ethics Committee of Fudan University Eye and ENT Hospital Review. Written informed consent to participate in this study was provided by the participants' legal guardian/next of kin. Written informed consent was obtained from the individual(s) for the publication of any potentially identifiable images or data included in this article.

## Author Contributions

All authors read and approved the final manuscript. XZ: study concept and design and supervision. ML and RW: data collection. RW: writing the manuscript. ML and XZ: critical revision of the manuscript. All authors analysis and interpretation of data.

## Conflict of Interest

The authors declare that the research was conducted in the absence of any commercial or financial relationships that could be construed as a potential conflict of interest.

## Publisher's Note

All claims expressed in this article are solely those of the authors and do not necessarily represent those of their affiliated organizations, or those of the publisher, the editors and the reviewers. Any product that may be evaluated in this article, or claim that may be made by its manufacturer, is not guaranteed or endorsed by the publisher.
